# Theoretical Model for Ostwald Ripening of Nanoparticles with Size-Linear Capture Coefficients

**DOI:** 10.3390/nano15221719

**Published:** 2025-11-13

**Authors:** Vladimir G. Dubrovskii, Egor D. Leshchenko

**Affiliations:** 1 Faculty of Physics, St. Petersburg State University, Universitetskaya Emb. 13B, 199034 St. Petersburg, Russia; 2 Submicron Heterostructures for Microelectronics, Research and Engineering Center RAS, Politekhnicheskaya Street, 26, 194021 St. Petersburg, Russia

**Keywords:** Ostwald ripening, kinetic equation, critical size, size distribution, scaling

## Abstract

The Ostwald ripening process in 3D and 2D systems has been studied in great detail over decades. In the application to surface nanoislands and nanodroplets, it is usually assumed that the capture coefficients of adatoms by supercritical nanoparticles of size s scale as $s^{\alpha }$, where the growth index α is smaller than unity. Here, we study theoretically the Ostwald ripening of 3D and 2D nanoparticles whose capture coefficients scale linearly with s. This case includes submonolayer surface islands that compete for the flux of highly diffusive adatoms upon termination of the material influx. We obtain analytical solutions for the size distributions using the Lifshitz–Slezov scaled variables. The distributions over size s and radius R are monotonically decreasing, and satisfy the normalization condition for different values of the Lifshitz–Slezov constant c. The obtained size distributions satisfy the Family–Vicsek scaling hypothesis, although the material influx is switched off. The model is validated by fitting the monotonically decreasing size distributions of Au nanoparticles that serve as catalysts for the vapor–liquid–solid growth of III-V nanowires on silicon substrates.

## 1. Introduction

The Ostwald ripening (OR) occurs in a late asymptotic stage of first-order phase transitions, where larger nuclei grow at the expense of smaller ones [1,2,3]. The OR process is usually considered at a time-independent total number of monomers (“mass” M) in a system [1,2,3,4,5,6,7,8,9,10,11,12,13], although it may also occur under weak material inputs [5,14]. When the critical size (number of monomers in the critical nucleus) $s_{*}$ enters the pre-existing size spectrum, subcritical nuclei with $s<s_{*}$ dissolve and increase supersaturation of a depleted mother phase, thus accelerating growth of supercritical nuclei with $s>s_{*}$. This is described by the term $1-{\left(s_{*}/s\right)}^{1/d}$ in the first-order kinetic equation for the SD, with d=3 or 2 as the dimension of 3D or 2D nuclei [1,2,5,12]. The form of this kinetic equation and the physical meaning suggest that the appropriately scaled SD depends only on the scaled size $x=s/s_{*}(\tau )$. Lifshitz and Slezov [1,2] obtained the universal scaled SD $F\left(x\right)\;$ in the specific case of diffusive decomposition of supersaturated solid solutions (d=3) at M=const, where the regular growth rate of nuclei (at $s_{*}/s\to 0)$ scales as $s^{1/3}$. Later on, the Lifshitz–Slezov (LS) method was successfully used for modeling the OR process in 2D and 3D systems for the power-law capture coefficients $\sigma \left(s\right)=s^{\alpha }$ with different growth indices α<1 [1,2,3,4,5,6,7,8,9,10,11,12,13,14], including the data of in situ monitoring by transmission electron microscopy (TEM) [4,7]. The classical LS spectra at α=0 are unimodal and tend to zero at x=0 and at the “blocking” point $x=x_{max}$ [1,2,5,6,8,9,10,11,12]. The shape of the LS spectra critically depends on the numerical value of a constant c (the LS constant) that arises due to separation of variables in the kinetic equation for the SD. The scaled SDs at any α are consistent with the normalization condition M=const only for $c\leq c_{max}(\alpha )$. The mass becomes discontinuous for $c>c_{max}(\alpha )$. In their first paper [1], Lifshits and Slezov presented the arguments for the well-defined choice of $c=c_{max}$. These arguments are definitely valid in the case of infinite initial spectra without a maximum nucleus. Later on, it was shown that other c may be relevant in a more complex situation of finite spectra having a maximum nucleus size $s_{max}$ [9,12]. Therefore, the LS solutions generally yield a family of solutions for the SD, where the correct choice of the LS constant depends on the prehistory of the system in the preceding earlier growth steps.

According to the LS theory, the scaled SDs in the OR stage acquire universal shapes. This is strongly reminiscent of the Family–Viscek (FV) scaling [15], which is observed in the SDs of surface islands in the pre-coalescence stage of their growth [16,17,18,19,20,21,22,23] and has far-reaching implications in areas far beyond epitaxial growth [24,25,26,27,28]. One may also recall the similarity considerations that lead to the universal SDs of coagulating aerosols described by the Smoluchowsky equation [29,30] and even more complex models with simultaneous coagulation and condensation [31], or similarity solutions of the Becker–Döring equations for reversible growth [32,33,34,35]. The universality of the scaled SDs is important from a fundamental viewpoint and for understanding and modeling particular systems [4,5,6,13,14,25,36,37,38,39,40]. The case of size-linear growth rates or capture coefficients is relevant for large enough 2D surface islands [19,21,22,23,24], vertical nanowires [25], other 1D structures [14], and aerosols [31], which often yield exact solutions for the universal SDs [24,29,31,32,34,40]. In the limit of infinitely large ratios of the adatom diffusion coefficient over the deposition rate, the asymptotically linear size dependence of the capture coefficients of epitaxial islands arises due to subtle correlations between island sizes and separation [19,20,21,22,23]. For surface islands, the capture coefficient $\sigma \left(s\right)\;$ is roughly proportional to the mean capture zone area A(s). Larger islands typically exhibit larger A(s), which lead to faster growth of these inlands. In the agglomeration regime, A(s) is proportional to s, which gives $\sigma \left(s\right)\sim A\left(s\right)\sim s$ [19,23]. In the vapor–liquid–solid growth of semiconductor nanowires, the size linearity of vertical growth rate is explained by surface diffusion of adatoms along the entire length of nanowire sidewalls. In this regime of the diffusion-induced nanowire growth, the capture rate is given by $\sigma \left(s\right)=A+s$, with A as a constant that describes direct impingement of the vapor flux onto the droplet surface [25]. All these growth regimes are distinctly different from the power-law $\sigma \left(s\right)=s^{\alpha }$ with α<1. However, the size-linear model $\sigma \left(s\right)=s$ has not been considered in the theory of OR to our knowledge. Our goal in this paper is to obtain the analytic SDs for 3D and 2D nanoparticles with size-linear capture coefficients in the OR stage upon termination of the material influx. We will show that these SDs are monotonically decreasing in the entire range of possible LS constants c from zero to d+1, in contrast to the previously obtained unimodal LS functions at α<1 [1,2,3,4,5,6,7,8,9,10,11,12,13,14]. It will also be demonstrated that these SDs satisfy a modified FV scaling hypothesis with the same sum rule for the island size, despite the fact that the nanoparticle density decreases in the OR stage. The model will be used to describe the SDs of Au nanodroplets on patterned SiO_x_/Si (111) substrates that are used as catalysts for the vapor–liquid–solid growth of semiconductor nanowires [25,41,42,43,44].

## 2. Model

We consider the SD $n\left(s,\tau \right)\;$over size s, which equals the number of monomers in the nucleus, with τ as a dimensionless time. In a closed system, upon termination of a monomer input, supersaturation tends to zero. Due to a steep exponential dependence of the Zeldovich nucleation rate on supersaturation, no new particles nucleate in the asymptotic OR stage [1,2]. The nanoparticle density N(τ), the constant mass M, and the average size$\;\bar{s}(\tau )$ are given by

(1) $$N\left(\tau \right)=\int_{0}^{\infty }dsn(s,\tau ),\;M=\int_{0}^{\infty }dssn\left(s,\tau \right)=const,\;\bar{s}(\tau )=\frac{M}{N(\tau )}\;.$$

Obviously, the average size can increase only due to a decreasing density, which is a typical feature of the OR process at M=const. The first-order kinetic equation for the SD is written as [14]:

(2) $$\frac{\partial n(s,\tau )}{\partial \tau }=-\frac{\partial }{\partial s}\left[W^{+}(s)\left(1-e^{dF/ds}\right)n(s,\tau )\right]$$

Here,

(3) $$W^{+}\left(s\right)=(1+\zeta )\sigma (s)$$

is the condensation rate, which contains a time-dependent supersaturation ζ(τ) and is proportional to a size-dependent capture coefficient σ(s). In the theory of OR, it is usually assumed that $\sigma \left(s\right)=s^{\alpha }$ [1,2,5,6,9,10,11,12,13,14], where the growth index α depends on the dimensionality of the nucleus and the mother phase, and the mechanism of material transport into the nucleus [14]. Below, we consider the size-linear capture coefficient

(4) $$\sigma \left(s\right)=s,$$

which is relevant for a wide range of systems as discussed above.

The term 1−exp(dF/ds) in Equation (2) describes the evaporation of monomers from nuclei. The free energy of forming the nucleus with size s in thermal units of $k_{B}T$ is given by [14]

(5) $$F\left(s\right)=as^{\mu }-\ln\left(1+\zeta \right)s.$$

The second term describes the energetically favorable decrease in chemical potential due to condensation. The first term describes the energetically unfavorable process of forming a nucleus surface. The parameter a equals the appropriately normalized surface energy γ: $a=r_{0}^{2}\gamma /\left(k_{B}T\right)$, with $r_{0}$ as the interatomic spacing in a condensed phase. It contains a large ratio of the characteristic surface energy over thermal energy and hence is much larger than unity. The surface area scales as $s^{1/2}$ for 2D and $s^{2/3}$ for 3D nucleus, hence μ=1/2 for d=2 and μ=2/3 for d=3. The nucleus formation energy given by Equation (5) reaches its maximum at the critical size

(6) $$s_{*}={\left[\frac{a}{2ln(1+\zeta )}\right]}^{2},\;\;d=2;\;\;s_{*}={\left[\frac{2a}{3ln(1+\zeta )}\right]}^{3},\;d=3.$$

The critical islands are in unstable equilibrium with a supersaturated mother phase; subcritical islands with $s<s_{*}$ tend to dissolve, while supercritical islands with $s>s_{*}$ tend to grow. From Equations (5) and (6), the derivative of the formation energy with respect to size equals $dF/ds=ln(1+\zeta )\left[{\left(s_{*}/s\right)}^{1/d-1}\right]$ for d=2 and 3. Using this in Equation (2), we obtain:

(7) $$\frac{\partial n(s,\tau )}{\partial \tau }=-\frac{\partial }{\partial s}\left\{\left[1+\zeta -{\left(1+\zeta \right)}^{U}\right]\sigma \left(s\right)n\left(s,\tau \right)\right\},\;U={\left(\frac{s_{*}}{s}\right)}^{1/d}.$$

Regular growth continues as long as $s_{*}\ll s$, corresponding to U→0. The asymptotic OR stage occurs at ζ→0, corresponding to the full depletion of a mother phase with monomers available for further growth. At  ζ→0, the critical size tends to infinity according to Equation (6). Therefore, one can use

(8) $$s_{*}={\left(\frac{a}{2\zeta }\right)}^{2},\;d=2;\;s_{*}={\left(\frac{2a}{3\zeta }\right)}^{3},\;d=3;\;{\left(1+\zeta \right)}^{U}=1+U\zeta .$$

This reduces Equation (2) to

(9) $$\frac{\partial n(s,\tau )}{\partial \tau }=-\zeta \frac{\partial }{\partial s}\left[\left(1-{\left(\frac{s_{*}}{s}\right)}^{1/d}\right)\sigma (s)n(s,\tau )\right],$$

which is the starting point for the theory of OR [1,2,3,4,5,6,7,8,9,10,11,12,13,14]. In our case, the size-linear capture coefficient is given by Equation (4).

## 3. The Lifshitz–Slezov Method

Following the LS method [1], we search for the SD in the form

(10) $$n\left(s,\tau \right)=\frac{f(x)}{s_{*}^{2}(\tau )},\;x=\frac{s}{s_{*}(\tau )}.$$

Such SD satisfies the normalization M=const provided that the function xf(x) is integrable from zero to infinity (or from zero to a locking point $x_{max}$ where $f\left(x\right)\;$ becomes zero [1,2]). Substituting this SD into Equation (9) with $\sigma \left(s\right)=s=s_{*}\left(\tau \right)x$ and using Equation (8) to express the unknown supersaturation through the critical size, we arrive at a separable equation, whose form depends on the nucleus dimensionality d. At d=3, we obtain

(11) $$\frac{1}{s_{*}^{2/3}}\frac{ds_{*}}{d\tau }\left[2f\left(x\right)+x\frac{df}{dx}\right]=\frac{2a}{3}\frac{d}{dx}\left[\left(x-x^{2/3}\right)f(x)\right].$$

Upon separation of variables, this is equivalent to

(12) $$\frac{1}{s_{*}^{2/3}}\frac{ds_{*}}{d\tau }=\frac{1}{c}\frac{2a}{3},$$

and

(13) $$2f\left(x\right)+x\frac{df}{dx}=c\frac{d}{dx}\left[\left(x-x^{2/3}\right)f(x)\right].$$

Here, c is the LS integration constant, which is the same in Equations (12) and (13). From Equation (12), the critical size scales as $\tau^{3}$:

(14) $$s_{*}={\left(\frac{2a}{9c}\tau \right)}^{3}.$$

Equation (12) for the scaled SD can be put in the form

(15) $$\frac{dlnf}{dx}=-\frac{1}{x}\frac{\left(2-c\right)x^{1/3}+2c/3}{\left(1-c\right)x^{1/3}+c}.$$

This can be analytically integrated at any c:

(16) $$f\left(x\right)=\frac{A}{x^{2/3}{\left[1+\frac{\left(1-c\right)}{c}x^{1/3}\right]}^{\left(4-c\right)/\left(1-c\right)}},$$

with A as the normalization amplitude that will be found later. This function describes infinite LS spectra at 0<c≤1 and finite spectra at 1<c<4 that tend to zero at the blocking point $x_{max}={\left[c/(c-1\right]}^{1/3}$. Both infinite and finite spectra satisfy the required normalization of constant mass at c<4. From Equation (16), it is clear that f(x) decreases faster than $x^{-2}$ at a large x when c≤1, corresponding to finite M in Equation (1). At c>1, $f\left(x\right)$ equals zero at $x>x_{max}$ [1]. At c=4, the distribution $A/x^{2/3}$ becomes non-physical, because it leads to discontinuous mass. At c=1, Equation (16) is reduced to

(17) $$f\left(x\right)=\frac{Ae^{-3x^{1/3}}}{x^{2/3}},\;c=1.$$

Repeating the same calculations in 2D space, we obtain

(18) $$s_{*}={\left(\frac{a\beta }{4c}\tau \right)}^{2},$$

Showing that the critical size of 2D islands scales as $\tau^{2}$. The equation for the LS scaling function is given by

(19) $$\frac{dlnf}{dx}=-\frac{1}{x}\frac{\left(2-c\right)x^{1/2}+c/2}{\left(1-c\right)x^{1/2}+c},$$

and has the solution

(20) $$f\left(x\right)=\frac{A}{x^{1/2}{\left[1+\frac{\left(1-c\right)}{c}x^{1/2}\right]}^{\left(3-c\right)/\left(1-c\right)}}.\;f\left(x\right)=\frac{Ae^{-2x^{1/2}}}{x^{1/2}},\;c=1.$$

This function describes infinite LS spectra at 0<c≤1 and finite spectra at 1<c<3 that tend to zero at the blocking point $x_{max}={\left[c/(c-1\right]}^{1/2}$. The total mass remains finite at c<3 and becomes infinite at c=3.

## 4. Density and Average Size

The time-dependent nanoparticle density $N\left(\tau \right)$ is obtained from the corresponding Equation (1). From Equation (10) for the scaled SD, it is clear that the density is inversely proportional to the critical size. The integration of $f\left(x\right)$ given by Equation (16) in the case of 3D case and Equation (20) in case of 2D x is easily performed and yields the same result

(21) $$N\left(\tau \right)=\frac{Ac}{s_{*}(\tau )}$$

for any c. Calculation of the average size is more easily performed using the normalized SD

(22) $$\varphi \left(s,\tau \right)=\frac{n(s,\tau )}{N(\tau )}=\frac{h(x)}{s_{*}(\tau )}.$$

From the corresponding Equation (1), it is clear that the average size is proportional to $s_{*}(\tau )$:

(23) $$\bar{s}\left(\tau \right)=s_{*}(\tau )\int_{0}^{\infty }dxxh(x)$$

The integral depends on the nucleus dimensionality. From Equations (10), (16) and (21), for 3D nuclei, we have

(24) $$h\left(x\right)=\frac{1}{cx^{2/3}{\left[1+\frac{\left(1-c\right)}{c}x^{1/3}\right]}^{\left(4-c\right)/\left(1-c\right)}}.$$

The integral in Equation (23) is calculated using

(25) $$\int_{0}^{\infty }\frac{dtt^{3}}{{\left(1+t\right)}^{\left(4-c\right)/\left(1-c\right)}}=\int_{0}^{1}dtt^{3}{\left(1-t\right)}^{\left(4-c\right)/\left(c-1\right)}=\frac{2{\left(1-c\right)}^{4}}{3c(1+2c)(2+c)}.$$

Here, the infinite upper integration limit that corresponds to c<1 is changed to unity for c>1. Hence, the normalization is the same for infinite and finite spectra h(x). Using Equation (25), we find:

(26) $$\int_{0}^{\infty }dxxh(x)=\frac{2c^{2}}{\left(1+2c)(2+c\right)},\;\bar{s}\left(\tau \right)={\frac{2c^{2}}{\left(1+2c)(2+c\right)}s}_{*}(\tau ).$$

Hence, the average size is smaller than critical for any c. For example, $\bar{s}\left(\tau \right)=(2/9)s_{*}(\tau )$ at c=1. From the normalization condition $N\left(\tau \right)\bar{s}\left(\tau \right)=M$ and Equations (21) and (26), we find the normalization amplitude in the form

(27) $$A=\frac{\left(1+2c)(2+c\right)}{2c^{3}}M.$$

From Equation (10), (20) and (21), for 2D nuclei we have

(28) $$h\left(x\right)=\frac{1}{cx^{1/2}{\left[1+\frac{\left(1-c\right)}{c}x^{1/2}\right]}^{\left(3-c\right)/\left(1-c\right)}}.$$

The integral in Equation (23) is calculated using

(29) $$\int_{0}^{\infty }\frac{dtt^{2}}{{\left(1+t\right)}^{\left(3-c\right)/\left(1-c\right)}}=\int_{0}^{1}dtt^{2}{\left(1-t\right)}^{\left(3-c\right)/\left(c-1\right)}=\frac{{\left(1-c\right)}^{3}}{2c(1+c)}.$$

As above, infinite and finite upper integration limits correspond to c<1 and c>1, respectively. This gives another relationship between the average and critical sizes in 2D space:

(30) $$\int_{0}^{\infty }dxxh(x)=\frac{c}{1+c\;},\;\bar{s}\left(\tau \right)=\frac{c}{1+c}s_{*}(\tau ).$$

As in the previous case, the average size is smaller than critical for any c. For example, $\bar{s}\left(\tau \right)=s_{*}(\tau )/2$ at c=1. The normalization amplitude of the SD is obtained from the condition $N\left(\tau \right)\bar{s}\left(\tau \right)=M$, Equations (21) and (30), and equals

(31) $$A=\frac{1+c}{c^{2}}M.$$

It is easy to check that

(32) $$N\left(\tau \right)=\frac{M}{\bar{s}\left(\tau \right)}$$

in all cases, as it should be in the OR process at M=const.

## 5. Size Distributions in Different Variables

Using the obtained normalization amplitudes given by Equation (27) for 3D nuclei and (31) for 2D nuclei, the size distributions over the natural size s (which equals the number of monomers in the nucleus) in the LS variables are obtained in the form

(33) $$n\left(s,\tau \right)=\frac{M}{s_{*}^{2}\left(\tau \right)}F\left(x\right),\;x=\frac{s}{s_{*}(\tau )}.$$

The scaled SD of 3D nuclei is given by

(34) $$F\left(x\right)=\frac{\left(1+2c)(2+c\right)}{2c^{3}}\frac{1}{x^{2/3}{\left[1+\frac{\left(1-c\right)}{c}x^{1/3}\right]}^{\left(4-c\right)/\left(1-c\right)}},\;F\left(x\right)=\frac{9}{2}\frac{e^{-3x^{1/3}}}{x^{2/3}},\;c=1.$$

For 2D nuclei, the scaled SD has the form

(35) $$F\left(x\right)=\frac{\left(1+c\right)}{c^{2}}\frac{1}{x^{1/2}{\left[1+\frac{\left(1-c\right)}{c}x^{1/2}\right]}^{\left(3-c\right)/\left(1-c\right)}},\;F\left(x\right)=2\frac{e^{-2x^{1/2}}}{x^{1/2}},\;c=1.$$

**Figure 1:**
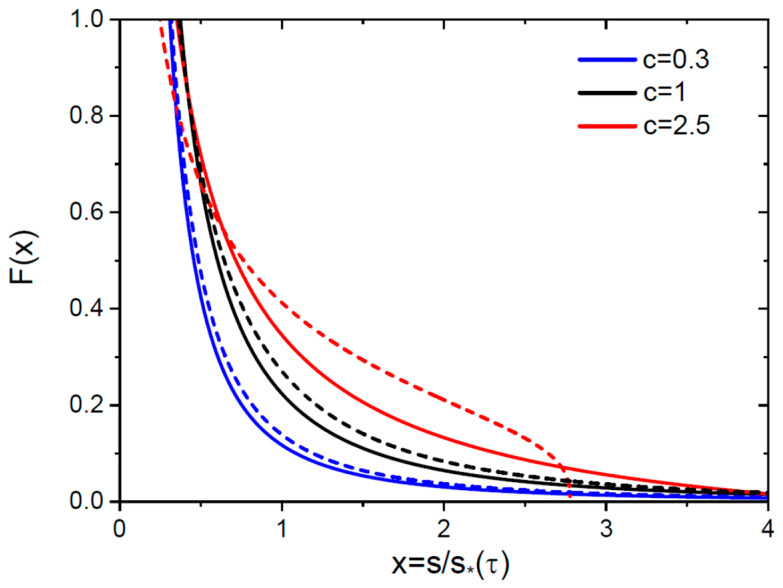
Scaled SDs $F={(s}_{*}^{2}/M)n$ versus scaled size $x=s/s_{*}(\tau )$ at three different c for 3D (solid lines) and 2D (dashed lines, same colors for each c) nuclei.

These SDs are relevant at 0<c<d+1, that is, smaller than 4 for 3D nuclei and smaller than 3 for 2D nuclei. In both cases, the scaled SDs are monotonically decreasing, and discontinuous at x=0 ($F\left(x\right)\sim x^{-2/3}$ at d=3 and $F\left(x\right)\sim x^{-1/2}$ at d=2). As discussed above, the scaled SDs are infinite at 0<c≤1 and finite at 1<c<d+1, with the maximum scaled size $x_{max}={\left[c/(c-1\right]}^{1/d}$. The scaled SDs satisfy the required sum rule

(36) $$\int_{0}^{\infty }dxxF\left(x\right)=1.$$

for any c<d+1. At c>1, the infinite limit of integration is changed to ${\;x}_{max}$. Figure 1 shows the infinite scaled SDs at c=0.3 and 1, and finite scaled SD at with a blocking point at c=2.5, obtained from Equation (34) at d=3 and (35) at d=2.

Let us now consider the radius distributions of nuclei, studied in the original works of Lifshitz and Slezov at α=1/3 [1,2]. According to the general rule [14], the radius distribution $g\left(R,\tau \right)$ should preserve the normalization:

(37) $$n\left(s,\tau \right)ds=g\left(R,\tau \right)dR,$$

The radius R and the critical radius $R_{*}\;$or, more generally, the linear sizes of a nanoparticle of any shape are related to the corresponding sizes s and $s_{*}\;$according to

(38) $$s={\left(\frac{R}{r}\right)}^{d},\;s_{*}={\left(\frac{R_{*}}{r}\right)}^{d}.$$

where r is an interatomic spacing. Using Equations (37) and (38) at d=3 in Equations (33) and (34), the distribution over radius of 3D nuclei is obtained in the form

(39) $$g\left(R,\tau \right)=\frac{r^{3}M}{R_{*}^{4}\left(\tau \right)}G\left(y\right),\;y=\frac{R}{R_{*}(\tau )},\;G\left(y\right)=\frac{3(1+2c)(2+c)}{2c^{3}}\frac{1}{{\left[1+\frac{\left(1-c\right)}{c}y\right]}^{\left(4-c\right)/\left(1-c\right)}},\;G\left(y\right)=\frac{27}{2}e^{-3y},\;c=1.$$

From Equation (14), the critical radius of 3D nuclei $R_{*}$ increases linearly with time:

(40) $$R_{*}\left(\tau \right)=r\frac{2a}{9c}\tau .$$

For 2D nuclei, the radius distribution is obtained from Equations (37) and (38) at d=2 and Equations (33) and (35). The result is given by

(41) $$g\left(R,\tau \right)=\frac{r^{2}M}{R_{*}^{3}\left(\tau \right)}G\left(y\right),\;y=\frac{R}{R_{*}(\tau )},\;\;G\left(y\right)=\frac{2(1+c)}{c^{2}}\frac{1}{{\left[1+\frac{\left(1-c\right)}{c}y\right]}^{\left(3-c\right)/\left(1-c\right)}},\;\;G\left(y\right)=4e^{-2y},\;c=1.$$

From Equation (18), the critical radius of 2D nuclei also scales linearly with time:

(42) $$R_{*}\left(\tau \right)=r\frac{a}{4c}\tau .$$

**Figure 2:**
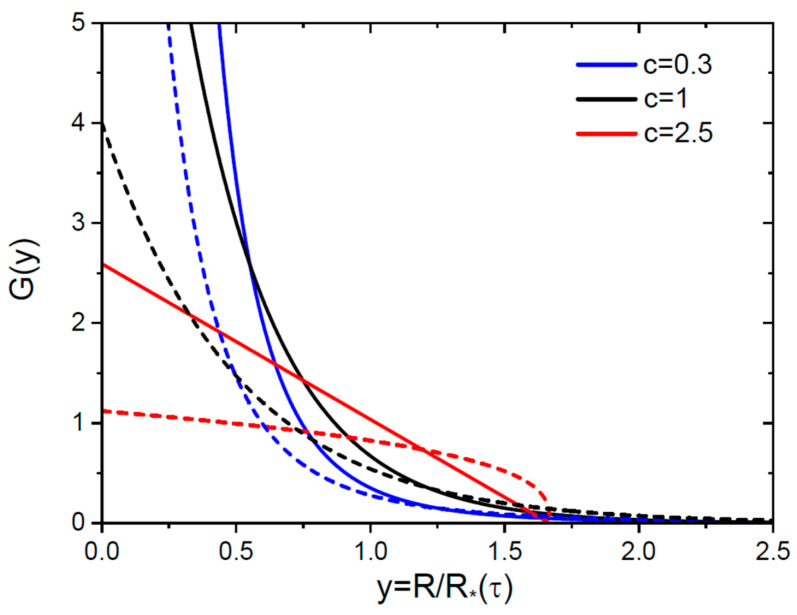
Scaled radius distributions G versus scaled radius $y=R/R_{*}(\tau )$ at three different c for 3D (solid lines) and 2D (dashed lines, same colors for each c) nuclei.

A common feature of the obtained radius distributions is that the discontinuities in the scaled distributions at x=y=0 are circumvented due to $ds/dR\sim s^{2/3}$ for 3D nuclei and $ds/dR\sim s^{1/2}$ for 2D nuclei. Consequently, the scaled functions $G\left(y\right)$ tend to a constant at y=0 and monotonically decrease with y, as shown in Figure 2 for the same parameters as above.

As mentioned above, the scaled LS distributions at α<1 [1,2,5,9,10,12] are unimodal. They vanish at y=0 and at the blocking point, and reach a maximum around y=1 [1]. Our results for the size-linear capture coefficients at α=1 are different, because the scaled SDs over size and radius are monotonically decreasing. This property of the linear model for σ(s) correlates with the earlier results of Refs. [31,32,34], where the monotonically decreasing scaled SDs at α=1 were obtained in different growth stages without OR, including the simplest exponential scaling function exp(−x). The monotonically decreasing scaled SDs at α=1, with discontinuity at x=0 for $F\left(x\right)$ physically mean that small subcritical nuclei remain most representative in the ensemble of nanoparticles. We note, however, that our solution yields unimodal SD over the so-called invariant size for which the regular growth rate becomes size-independent [12,14]. This invariant size is commonly used in nucleation and growth theory because the time-invariant SD of nanoparticles in the regular growth stage significantly simplifies mathematical computations and analysis of experimental data [14]. At $ds/d\tau \propto \sigma \left(s\right)=s$, the invariant size ρ is given by

(43) ρ=lns,

because dρ/dτ∝(dρ/ds)(ds/dτ)=1. Using ds/dρ=s for the “invariant” SD such that $n\left(s,\tau \right)ds=\psi \left(\rho ,\tau \right)d\rho$, we obtain:

(44) $$\psi \left(\rho ,\tau \right)=sn(s,\tau ).$$

Using Equation (33) for n(s,τ), the distribution over the invariant size of 3D nuclei becomes

(45) $$\psi \left(\rho ,\tau \right)=\frac{M}{s_{*}\left(\tau \right)}H\left(y\right),\;y=\frac{R}{R_{*}(\tau )}=e^{\left[\rho -\rho_{*}\left(\tau \right)\right]/d},$$

where $\rho_{*}\left(\tau \right)$ is the critical invariant size.

From Equation (34), we obtain the scaled invariant SD of 3D nuclei:

(46) $$H\left(y\right)=\frac{\left(1+2c)(2+c\right)}{2c^{3}}\frac{y}{{\left[1+\frac{\left(1-c\right)}{c}y\right]}^{\left(4-c\right)/\left(1-c\right)}},\;H\left(y\right)=\frac{9}{2}ye^{-3y},\;c=1.$$

Equation (35) yields the scaled invariant SD of 2D islands in the form

(47) $$H\left(y\right)=\frac{\left(1+c\right)}{c^{2}}\frac{y}{{\left[1+\frac{\left(1-c\right)}{c}y\right]}^{\left(3-c\right)/\left(1-c\right)}},\;H\left(y\right)=2ye^{-2y},\;c=1.$$

**Figure 3:**
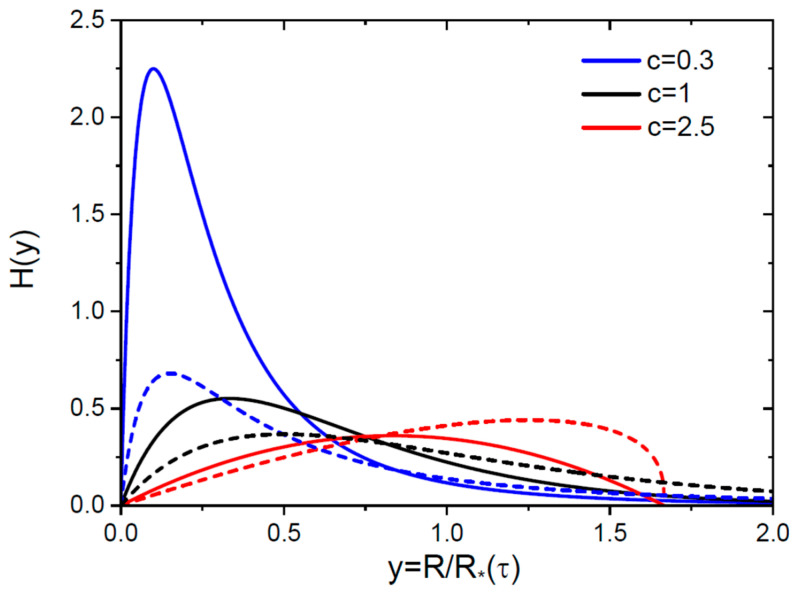
Scaled invariant distributions H versus scaled radius $y=R/R_{*}(\tau )$ at three different c shown in the legend for 3D (solid lines) and 2D (dashed lines, same colors for each c) nuclei.

These $H\left(y\right)$ scale as y at y→0 for any c and d. These scaling functions are shown in Figure 3 for the same c as above. The distributions at c=2.5 resemble the classical LS shapes [1,2,5,6,7,8,9,10,11,12,13,14], because they are unimodal and tend to zero at y=0 and at the blocking point $y=y_{max}$. The infinite SDs at c=0.3 and 1 are unimodal, but different from the usual LS shapes with the blocking points.

The FV scaling considered widely in the theory of 2D surface growth is formulated as follows [15,16,17,18,19,20,21,22,23,24]. In the limit of infinitely large ratios of the adatom diffusion coefficient over the deposition rate I, the island SD over s is expected to have the form $n\left(s,\tau \right)=(\theta /{\bar{s}}^{2})F_{0}(z)$ for all but very short times. Here, θ=Iτ is the surface coverage, $\bar{s}(\tau )$ is the average size and $F_{0}(z)$ is a universal function of the scaled size $z=s/\bar{s}(\tau )$. This type of scaling was studied based on the rate equations for irreversible growth (with the critical size one) under a time-independent deposition rate [18,19,22,23,24]. In this case, both island density and average size increase with time. Due to $N\left(\tau \right)\bar{s}\left(\tau \right)=\theta =I\tau$, any FV scaling function should satisfy the two sum rules for the island density and size. Due to subtle correlations between the island size and separation, the capture coefficients are much more complex than linear, but become asymptotically linear at large s [19,21,22,23]. The simplified linear model $\sigma \left(s\right)=s$ yields, however, a monotonically decreasing FV scaling function that satisfies both sum rules [24]. The OR process considered here is very different from irreversible growth, and occurs upon the flux termination. The mass M equals the surface coverage θ for 2D islands, but it stays constant rather than increases linearly with time. At M=const, the average size increases only due to the decrease in the island density that occurs due to dissolution of smaller islands into monomers. However, our analysis shows that the FV scaling hypothesis holds in the OR stage.

Indeed, using Equation (26) or (30) for the average size of 3D or 2D islands, the LS representation of the scaled SD given by Equation (33) can equivalently be presented in the FV form:

(48) $$n\left(s,\tau \right)=\frac{M}{{\bar{s}}^{2}\left(\tau \right)}F_{0}\left(z\right),\;z=\frac{s}{\bar{s}(\tau )}.$$

The FV scaling function for 3D islands is obtained from Equation (34) for F(x), re-written in terms of z:

(49) $$F_{0}\left(z\right)=\frac{1}{c}\frac{\alpha_{3}^{1/3}}{z^{2/3}{\left[1+\frac{\left(1-c\right)}{c}{\left(\alpha_{3}z\right)}^{1/3}\right]}^{\left(4-c\right)/\left(1-c\right)}},\;\;\alpha_{3}=\frac{2c^{2}}{\left(1+2c)(2+c\right)}\;F_{0}\left(z\right)={\left(\frac{2}{9}\right)}^{1/3}\frac{e^{-{\left(6z\right)}^{1/3}}}{z^{2/3}},\;c=1.$$

In 2D space, Equation (35) for F(x) yields the FV scaling function of the form

(50) $$F_{0}\left(z\right)=\frac{1}{c}\frac{\alpha_{2}^{1/2}}{z^{1/2}{\left[1+\frac{\left(1-c\right)}{c}{\left(\alpha_{2}z\right)}^{1/2}\right]}^{\left(3-c\right)/\left(1-c\right)}},\;\;\alpha_{2}=\frac{c}{1+c}\;F_{0}\left(z\right)=\frac{e^{-\sqrt{2z}}}{\sqrt{2z}},\;c=1.$$

**Figure 4:**
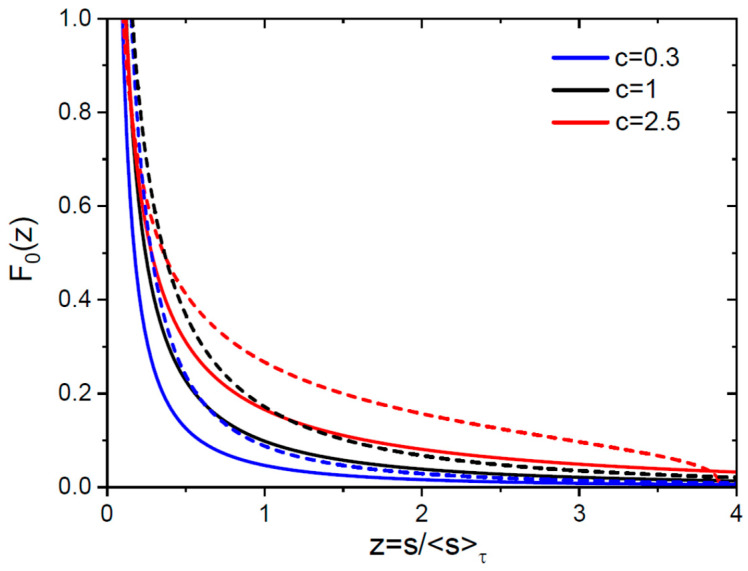
Scaled SDs $F_{0}=({\bar{s}}^{2}/M)n$ versus scaled size $x=s/\bar{s}(\tau )$ at three different c for 3D (solid lines) and 2D (dashed lines, same colors for each c) islands.

These scaling functions satisfy the sum rule for the average island size

(51) $$\int_{0}^{\infty }dzzF_{0}\left(z\right)=1$$

for any c and d. The time dependencies of the average size are given by Equation (14) for 3D islands and (18) for 2D islands, while the density is related to the average size by Equation (32) in both cases. The FV scaling functions, shown in Figure 4 for the same parameters as above, are not very different from the LS scaling functions in Figure 1. This is not surprising, because they are obtained by the simple transformation of variables from x to z.

## 6. Theory and Experiment

From the early days of nanowire physics and technology [41], Au has been used as catalyst for the vapor–liquid–solid growth of vertical nanowires of elemental [41] and III-V [25,42,43] semiconductors. To fabricate size-uniform nanowires in regular arrays, Au nanodroplets are positioned in the lithographically defined openings in an oxide or nitride mask on a substrate, often Si(111) [44,45]. It is important therefore to understand and control the radius distribution of Au droplets on SiO_x_/Si(111) prior to the nanowire growth [44,45]. In Ref. [45] the Au droplet dynamics on SiO_x_/Si(111) was monitored in situ using environmental TEM. Au was deposited onto the substrate at a low temperature, and then the temperature was increased up to ~ 800 °C to facilitate surface diffusion, formation of Au droplets, and filling the openings in the oxide layer. It was found that the droplet dynamics on the SiO_x_/Si(111) surface consist of distinctly different steps involving different processes. The 3D droplets are immovable at the beginning and participate in the OR process. In this step, the total volume of Au droplets is kept constant; smaller droplets disappear and larger droplets grow in size. The droplet density gradually decreases, and their average radius increases linearly with time. These features are typical for the classical OR process at a constant mass. In the next step, Au droplets start to move and coagulate. This process is complicated by the presence of an additional influx of Au adatoms from the surrounding substrate area. Finally, large droplets migrate to the openings, dissolve at a certain distance from the boundaries, Au adatoms migrate to the pinholes, and the droplets reappear in the openings.

**Figure 5:**
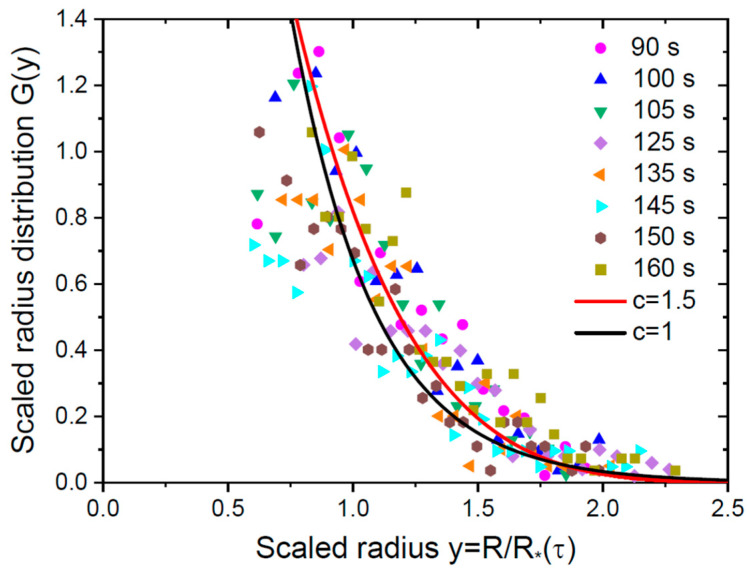
Scaled radius distributions of Au nanodroplets $G\propto R_{*}^{4}g\;$versus the scaled radius $y=R/R_{*}(\tau )$ at different times shown in legend, obtained from the data of Ref. [45] (symbols). Lines show the fits by Equation (39) at c=1 and 1.5.

Figure 5 shows the scaled radius distributions of Au nanodroplets in the LS variables. The scaled distributions are obtained from the radius histograms of Ref. [45] at different annealing times from 90 s to 160 s, corresponding to the OR of immovable Au droplets at a constant mass. The migration of droplets and their binary coagulation cannot be described within the current model. This sets the upper limit for the annealing time of 160 s where these processes start. The histograms were obtained from the same sample size of 20 µm × 20 µm. We can clearly see the monotonically decreasing shape of the scaled SDs despite the scattered data, at least for large enough droplets observed in TEM. These SDs are well-fitted by Equation (39) for the LS radius distribution at c≅1, as demonstrated by the curves at c=1 and 1.5 in the figure. We saw earlier that the values of c around unity yield close-to-exponential LS curves. Therefore, the fitting values of c~1 are chosen to match the exponential SDs in the regular growth stage before the OR [14] and the in the coagulation stage after the OR [30]. The unknown critical radii are calculated from the measured average radii using Equation (26). We may thus conclude that the capture rates of these 3D droplets are approximately linear in s, because other LS spectra at any α<1 yield the unimodal SDs that are not observed experimentally. We suspect that the linear behavior of σ(s) at large s is related to the high diffusivity of Au adatoms on the oxide surface, similarly to Refs. [19,23]. The microscopic analysis of the σ(s) dependence in this system requires additional studies and will be presented elsewhere.

## 7. Conclusions

In summary, we have obtained the analytic LS spectra in the OR stage of 3D and 2D nanoparticles with size-linear capture coefficients $\sigma \left(s\right)=s$. It has been shown that the scaled SDs over s are monotonically decreasing and discontinuous at x=0. The sum rule for the constant total mass is satisfied for any value of the LS constant c between zero and d+1, with d=3 for 3D and 2 for 2D particles. The scaled SDs are infinite at c≤1 and finite at c>1, with the blocking point at $x_{max}={\left[c/(c-1\right]}^{1/d}$. The scaled LS SDs over the radius (or linear size of anisotropic crystal islands) are monotonically decreasing, but tend to a constant at y=0. The unimodal SDs that vanish at y=0 are observed only for the invariant size variable for which the regular growth rate is size-independent. It has been demonstrated that the LS spectra can equivalently be presented in the FV scaling form. Therefore, the FV scaling hypothesis works well also in the OR stage, although it was originally proposed for epitaxial islands growing under a constant deposition flux with the critical size one. The obtained results should be useful for modeling and analysis of the monotonically decreasing SDs in the OR stage, as demonstrated by the example of Au nanodroplets on SiO_x_/Si(111) substrates. The 2D surface islands in different material systems and morphologies require a separate study. It will be interesting to consider a more complex growth scenario for the OR of 2D islands, where the scaled capture coefficient have the form $\sigma (s)/\bar{\sigma }=C(z)$ with the asymptotic linear increase $C\left(z\right)\sim z$ at large z [19,23]. This case corresponds to a wide range of practically important epitaxial systems with very high ratios of the adatom diffusion coefficient over the deposition rate. Scaling of the capture coefficient and the decomposition rate of subcritical nuclei with the average size should lead to a scaling solution for the SD, but its form is expected to be very different from the classical LS shapes. The physical choice of the LS constant c should depend on the shape of the initial SD that is determined in an early growth stage preceding the OR process. We plan to study these questions in a future paper.

## Data Availability

The original contributions presented in this study are included in the article. Further inquiries can be directed to the corresponding author.

## References

[1] Lifshitz I.M., Slezov V.V. (1959). Kinetics of diffusive decomposition of supersaturated solid solutions. Sov. Phys. JETP.

[2] Lifshitz I.M., Slezov V.V. (1961). The kinetics of precipitation from supersaturated solid solutions. J. Phys. Chem. Solids.

[3] Galvani N., Pitois O., Cohen-Addad S. (2025). Coarsening of bubble assemblies: From dry foams to dilute bubbly liquids. J. Col. Interface Sci..

[4] Geng L., Liu Q., Chen J., Jia P., Ye H., Yan J., Zhang L., Tang Y., Huang J. (2022). In situ observation of electrochemical Ostwald ripening during sodium deposition. Nano Res..

[5] Kukushkin S.A., Osipov A.V. (2007). Thin-film condensation processes. Phys. Usp..

[6] Voorhees P.W. (1985). The theory of Ostwald ripening. J. Stat. Phys..

[7] Simonsen S.B., Chorkendorff I., Dahl S., Skoglundh M., Sehested J., Helveg S. (2011). Ostwald ripening in a Pt/SiO_2_ model catalyst studied by in situ TEM. J. Catal..

[8] Koroleva M.Y., Yurtov E.V. (2021). Ostwald ripening in macro- and nanoemulsions. Russ. Chem. Rev..

[9] Giron B., Meerson B., Sasorov P.V. (1998). Weak selection and stability of localized distributions in Ostwald ripening. Phys. Rev. E.

[10] Behrens M.A., Franzén A., Carlert S., Skantze U., Lindfors L., Olsson U. (2025). On the Ostwald ripening of crystalline and amorphous nanoparticles. Soft Matter.

[11] Streets A.M., Quake S.R. (2010). Ostwald ripening of clusters during protein crystallization. Phys. Rev. Lett..

[12] Dubrovskii V.G., Kazansky M.A., Nazarenko M.V., Adzhemyan L.T. (2011). Numerical analysis of Ostwald ripening in two-dimensional systems. J. Chem. Phys..

[13] Slezov V.V. (2009). Kinetics of First-Order Phase Transitions.

[14] Dubrovskii V.G. (2014). Nucleation Theory and Growth of Nanostructures.

[15] Vicsek T., Family F. (1984). Dynamic scaling for aggregation of clusters. Phys. Rev. Lett..

[16] Evans J.W., Thiel P.A., Bartelt M.C. (2006). Morphological evolution during epitaxial thin film growth: Formation of 2D islands and 3D mounds. Surf. Sci. Rep..

[17] Einax M., Dieterich W., Maass P. (2013). Colloquium: Cluster growth on surfaces: Densities, size distributions, and morphologies. Rev. Mod. Phys..

[18] Bartelt M.C., Evans J.W. (1992). Scaling analysis of diffusion-mediated island growth in surface adsorption processes. Phys. Rev. B.

[19] Bartelt M.C., Evans J.W. (1996). Exact island-size distributions for submonolayer deposition: Influence of correlations between island size and separation. Phys. Rev. B.

[20] Bales G.S., Chrzan D.C. (1994). Dynamics of irreversible island growth during submonolayer epitaxy. Phys. Rev. B.

[21] Gibou F.G., Ratsch C., Caflisch R.E. (2003). Capture numbers in rate equations and scaling laws for epitaxial growth. Phys. Rev. B.

[22] Vvedensky D.D. (2000). Scaling functions for island-size distributions. Phys. Rev. B.

[23] Korner M., Einax M., Maass P. (2012). Capture numbers and island size distributions in models of submonolayer surface growth. Phys. Rev. B.

[24] Dubrovskii V.G. (2025). A general solution to the continuum rate equation for island-size distributions: Epitaxial growth kinetics and scaling analysis. Nanomaterials.

[25] Dubrovskii V.G., Berdnikov Y., Schmidtbauer J., Borg M., Storm K., Deppert K., Johansson J. (2016). Length distributions of nanowires growing by surface diffusion. Cryst. Growth Des..

[26] Fujimoto K., Hamazaki R., Kawaguchi Y. (2020). Family-Vicsek scaling of roughness growth in a strongly interacting Bose gas. Phys. Rev. Lett..

[27] Bhakuni D.S., Lev Y.B. (2024). Dynamic scaling relation in quantum many-body systems. Phys. Rev. B.

[28] Aditya S., Roy N. (2024). Family-Vicsek dynamical scaling and Kardar-Parisi-Zhang-like superdiffusive growth of surface roughness in a driven one-dimensional quasiperiodic model. Phys. Rev. B.

[29] Friedlander S.K. (1960). Similarity considerations for the particle size spectrum of a coagulating, sedimenting aerosol. J. Meteorol..

[30] Piskunov V.N., Golubev A.I., Goncharov E.A., Ismailova N.A. (1997). Kinetic modeling of composite particles coagulation. J. Aerosol Sci..

[31] Aloyan A.E., Egorov V.D., Marchuk G.I., Piskunov V.N. (1992). Aerosol formation mathematical modelling with consideration for condensation kinetics. Rus. J. Num. Anal. Math. Model..

[32] Brilliantov N.V., Krapivsky P.L. (1991). Non-scaling and source-induced scaling behaviour in aggregation models of movable monomers and immovable clusters. J. Phys. A Math. Gen..

[33] Wattis J.A.D. (2004). Similarity solutions of a Becker-Döring system with time-dependent monomer input. J. Phys. A Math. Gen..

[34] Dubrovskii V.G. (2025). Analytical solutions of the Becker–Doring equations with size-linear rate constants: Transition from limited to infinite growth regime. J. Phys. A Math. Theor..

[35] King J.R., Wattis J.A.D. (2002). Asymptotic solutions of the Becker–Döring equations with size-dependent rate constants. J. Phys. A Math. Gen..

[36] Petrov P.P., Miller W., Rehse U., Fornari R. (2011). A new method for calculation of island-size distribution in submonolayer epitaxial growth. Appl. Math. Model..

[37] González D.L., Camargo M., Sánchez J.A. (2018). Island size distribution with hindered aggregation. Phys. Rev. E.

[38] Álvarez-Cuartas J.D., González-Cabrera D.L., Camargo M. (2024). Epitaxial growth in one dimension. J. Phys Cond. Matter.

[39] Dubrovskii V.G. (2025). Family-Vicsek scaling in classical nucleation theory. Phys. Rev. E.

[40] Tomellini M., De Angelis M. (2025). Fokker-Planck equation for the crystal-size probability density in progressive nucleation and growth with application to lognormal, Gaussian and gamma distributions. J. Cryst. Growth.

[41] Wagner R.S., Ellis W.C. (1964). Vapor-liquid-solid mechanism of single crystal growth. Appl. Phys. Lett..

[42] Dalacu D., Kam A., Austing D.G., Wu X., Lapointe J., Aers G.C., Poole P.J. (2009). Selective-area vapour–liquid–solid growth of InP nanowires. Nanotechnology.

[43] Kelrich A., Calahorra Y., Greenberg Y., Gavrilov A., Cohen S., Ritter D. (2013). Shadowing and mask opening effects during selective-area vapor-liquid-solid growth of InP nanowires by metalorganic molecular beam epitaxy. Nanotechnology.

[44] Hijazi H., Leroy F., Monier G., Grégoire G., Gil E., Trassoudaine A., Dubrovskii V.G., Castelluci D., Goktas N.I., LaPierre R.R. (2020). Dynamics of Au droplet formation on SiO_2_/Si(111) surface. J. Phys. Chem. C.

[45] Panciera F., Dubrovskii V.G., Reznik R.R., Cirlin G.E., Harmand J.C. In situ monitoring and modeling of the Au droplet dynaimcs on patterned SiO_x_/Si(111) substrate. ACS Nano.

